# Generation of hyper-entanglement in polarization/energy-time and discrete-frequency/energy-time in optical fibers

**DOI:** 10.1038/srep09195

**Published:** 2015-03-17

**Authors:** Shuai Dong, Lingjie Yu, Wei Zhang, Junjie Wu, Weijun Zhang, Lixing You, Yidong Huang

**Affiliations:** 1 Tsinghua National Laboratory for Information Science and Technology, Department of Electronic Engineering, Tsinghua University, Beijing, 100084, China; 2 State Key Laboratory of Functional Materials for Informatics, Shanghai Institute of Microsystem and Information Technology, Chinese Academy of Sciences, Shanghai 200050, China

## Abstract

In this paper, a generation scheme for telecom band hyper-entanglement is proposed and demonstrated based on the vector spontaneous four wave mixing (SFWM) processes in optical fibers. Two kinds of two-photon states are generated, one is hyper-entangled in the degree of freedoms (DOFs) of energy-time and polarization, the other is hyper-entangled in DOFs of energy-time and discrete-frequency. Experiments of Franson-type interference, two-photon interference under non-orthogonal polarization bases and spatial quantum beating are realized to demonstrate the entanglement in energy-time, polarization and frequency, respectively. This scheme provides a simple way to realize telecom band hyper-entanglement, which has potential for large geographic-scale applications of quantum communication and quantum information over optical fibers.

With entanglement in more than one degrees of freedom (DOFs) of photons, hyper-entangled photon pairs^1^ are important resources in quantum information applications including quantum super-dense coding^2^,^3^ and quantum key distribution^4^. Previous schemes for hyper-entanglement generation were usually based on spontaneous parametric down-conversion (SPDC) in nonlinear crystals^5^. Polarization, momentum and energy-time are most commonly used DOFs of photons in hyper-entanglement based on SPDC. In recent years, as the development of quantum engineering, entangled photon-pair generation schemes based on spontaneous four wave mixing (SFWM) in optical fibers have attracted much attention. These schemes support entanglement generation at low loss transmission window of silica optical fibers, based on technologies of optical communication and networks^6^,^7^,^8^,^9^,^10^,^11^,^12^. Recently, the generation of energy-time entangled photon pairs is realized in optical fibers^13^, which has great potential for large geographic-scale quantum information applications, such as long distance quantum key distribution^14^,^15^, thanks to its robustness to environment variations. It can be expected that hyper-entanglement in energy-time and other DOFs would further extend the applications of entangled photon pairs over optical fibers.

In this paper, we propose and demonstrate a scheme to generate 1.5 μm hyper-entangled photon pairs in optical fibers. Two kinds of hyper-entangled two-photon states are realized, one is hyper-entangled in DOFs of energy-time and polarization, the other is hyper-entangled in DOFs of energy-time and discrete-frequency. This scheme is based on the indistinguishability of the two vector SFWM processes in optical fibers, which are stimulated simultaneously by two pump lights with orthogonal polarizations and different frequencies. The experiments of Franson-type interference^16^, two-photon interference under non-orthogonal polarization bases, and spatial quantum beating^17^ are realized to demonstrate the entanglements in the generated photon pairs in the DOFs of energy-time, polarization and discrete-frequency, respectively.

## Results

### Principle of hyper-entanglement generation based on vector SFWM in optical fibers

**Figure 1:**
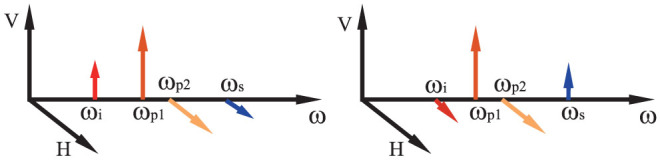
Vector SFWM processes stimulated by two pump lights with orthogonal polarizations and different frequencies for the hyper-entanglement generation. H and V denote two orthogonal polarization directions (H: Horizontal direction, V: Vertical direction). The proposed scheme for the hyper-entanglement generation is based on the coherent superposition of the two vector SFWM processes.

The SFWM is a kind of nonlinear processes caused by the *χ*^(3)^ nonlinearity, in which two pump photons are annihilated, while two photons are generated simultaneously. Traditionally, the photon with higher frequency is named as signal photon, and the other photon is named as idler photon. The energy conservation is satisfied in the SFWM processes, leading to the frequency relation of *ω_p_*_1_ + *ω_p_*_2_ = *ω_s_* + *ω_i_*, where *ω_p_*_1_, *ω_p_*_2_, *ω_s_*, and *ω_i_* denote the frequencies of the two pump photons, the signal photon and the idler photon, respectively. For an isotropic medium, the nonzero elements of the third order nonlinear tensor *χ*^(3)^ are 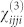, 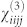, 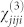, where *i*, *j* ∈ {*x*, *y*, *z*}, *x*, *y* and *z* are three orthogonal coordinate directions. In optical fibers, the 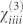, *i* ∈ {*x*, *y*} supports scalar SFWM processes, in which the annihilated pump photons and generated signal and idler photons are all polarized in the same direction. While, the 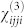, 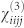, and 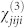, *i*, *j* ∈ {*x*, *y*}, *i* ≠ *j*, may support vector SFWM processes. Two kinds of vector SFWM processes are shown in Fig. 1, in which the annihilated two pump photons are polarized in two orthogonal directions, the generated signal and idler photons are also polarized orthogonally. H and V in the figure denote two orthogonal polarization directions (H: Horizontal direction, V: Vertical direction) in the fiber.

In the proposed schemes for the hyper-entanglement generation in optical fibers, two orthogonally polarized continuous wave (CW) pump lights with different frequencies are injected into the optical fiber to stimulate the two vector SFWM processes simultaneously. The two processes are indistinguishable and coherently superposed, which is the basis for the hyper-entanglement generation. In each vector SFWM process shown in Fig. 1, the signal and idler photons are generated simultaneously, ensuring the property of energy-time entanglement. The two-photon states generated by the two vector SFWM processes can be expressed as^8^
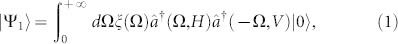

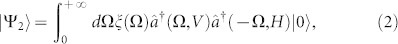


where |Ψ_1_〉 and |Ψ_2_〉 are the states for the left and right processes in Fig. 1, respectively. Ω ≡ (*ω_p_*_1_ + *ω_p_*_2_)/2 − *ω_i_* = *ω_s_* − (*ω_p_*_1_ + *ω_p_*_2_)/2, is the frequency detuning, where *ω_p_*_1_, *ω_p_*_2_, *ω_s_* and *ω_i_* are the frequencies of the two pump lights, the signal photon and idler photon, respectively. In an optical fiber without birefringence, the amplitudes of the two-photon states generated by the two vector SFWM processes are the same, which are denoted by *ξ*(Ω).

Equation (1) and (2) show that in each vector SFWM process, the two photons in a pair are polarized orthogonally. To generate the hyper-entanglement in DOFs of energy-time and polarization, a proper optical filter system should be applied to route the generated photons to two paths by their frequencies. In each path, the polarizations of the generated photons are not determined due to the indistinguishability of the two vector SFWM processes, leading to the polarization entanglement generation. The central frequencies of the filters for the signal and idler photons can be denoted by *ω_s_*_0_ and *ω_i_*_0_, respectively, which satisfy the relation of *ω_s_*_0_ + *ω_i_*_0_ = *ω_p_*_1_ + *ω_p_*_2_. The filter bandwidths for the signal and idler photons are the same and denoted by *δ*. If the bandwidth *δ* is narrow, the two-photon state at the two paths after the optical filter system can be expressed as
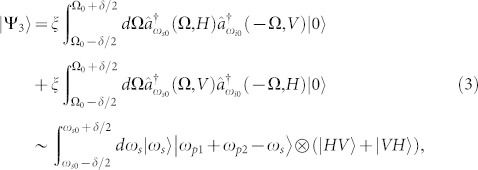
where, 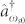 and 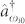 denote the creation operators at the two paths for the signal and idler photons, respectively. Ω_0_ is defined by Ω_0_ ≡ (*ω_p_*_1_ + *ω_p_*_2_)/2 − *ω_i_*_0_ = *ω_s_*_0_ − (*ω_p_*_1_ + *ω_p_*_2_*)*/2. *ξ*(Ω) is approximated to a constant *ξ* = *ξ*(Ω). The state |Ψ_3_〉 shows hyper-entanglement in DOFs of energy-time and polarization.

To generate hyper-entanglement in DOFs of energy-time and discrete-frequency, a polarization beam splitter should be used to route the generated photons by the two vector SFWM processes to two paths by their polarizations. The polarization relation of signal and idler photons in the vector SFWM processes ensures that the two generated photons in the same process would exit via the two paths separately. While, in each path the frequencies of the generated photons are not determined due to the indistinguishability of the two vector SFWM processes, leading to the frequency entanglement generation. Especially, if the frequencies of the signal and idler photons are defined by narrow band optical filters at *ω_s_*_0_ and *ω_i_*_0_ with bandwidths of *δ*, the discrete-frequency entanglement can be generated. If the bandwidth *δ* is narrow, the two-photon state can be expressed as
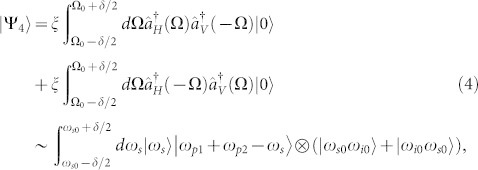
where, 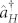 and 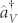 denote the creation operators at the two paths for the photons polarized in H and V directions, respectively. Ω_0_ is defined by Ω_0_ ≡ (*ω_p_*_1_ + *ω_p_*_2_)/2 − *ω_i_*_0_ = *ω_s_*_0_ − (*ω_p_*_1_ + *ω_p_*_2_)/2 and *ξ*(Ω) is approximated to a constant *ξ* = *ξ*(Ω). The state |Ψ_4_〉 shows hyper-entanglement in DOFs of energy-time and discrete-frequency.

### Hyper-entanglement in energy-time and polarization

**Figure 2:**
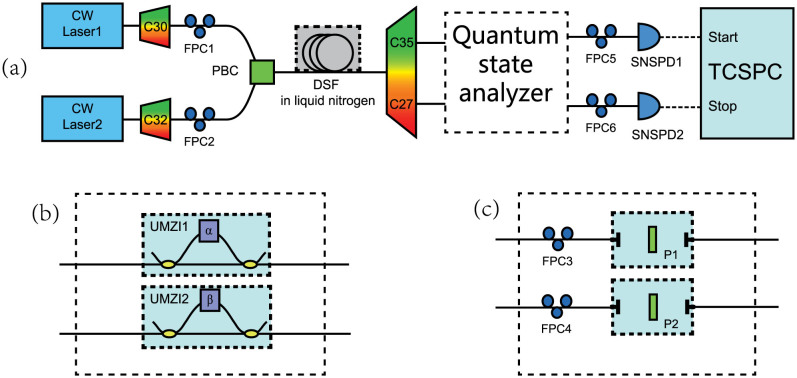
The experiment setup for the demonstration of hyper-entanglement in energy-time and polarization. (a) Two CW pump lights with orthogonal polarizations and different frequencies are combined by a PBC, then injected into a piece of DSF to stimulate the vector SFWM processes, generating correlated signal and idler photons with orthogonal polarizations. The signal and idler photons are routed to two paths according to their frequencies by an optical filter module realized by cascaded DWDMs. The generated photon pairs are measured by the quantum states analyzer for specific entanglement and two SNSPDs. The measurement results are recorded by a TCSPC module. (b) The quantum state analyzer for energy-time entanglement, composed of two UMZIs. (c) The quantum state analyzer for polarization entanglement, composed of two fiber polarization controllers (FPCs) and two rotatable polarizers (Ps).

The experiment setup for the demonstration of hyper-entanglement in energy-time and polarization is shown in Fig. 2. The two pump lights are generated by two CW lasers (Agilent 81980 A and Bookham TL5000DCJ), under wavelengths of 1551.80 nm (C32, ITU channel 32) and 1553.33 nm (C30, ITU channel 30), respectively. The linewidths of the two lasers are 100 kHz (Agilent 81980A) and 5 MHz (Bookham TL5000DCJ), respectively. Each of the pump light passes through an optical filter module to suppress noise photons at the signal and idler frequencies. The two pump lights are combined under orthogonal polarization directions by a polarization beam combiner (PBC). A piece of dispersion shifted fiber (DSF) is used as the nonlinear medium, which is about 500 meters in length. The DSF is cooled by liquid nitrogen bath to suppress the noise photons generated by the spontaneous Raman scattering^18^. Correlated signal and idler photons are generated in the DSF by the vector SFWM processes with orthogonal polarization directions.

To generate hyper-entanglement in energy-time and polarization, the generated photons are routed to two paths according to their frequencies by an optical filter module composed of cascaded commercial density wavelength division multiplexers (DWDMs). The central wavelength and full width at half maximum (FWHM) of the filter for the signal photons are 1549.32 nm (C35, ITU channel 35) and 118 GHz, respectively. Those for the idler photons are 1555.75 nm (C27, ITU channel 27) and 139 GHz, respectively [see Methods for more details]. Then, the selected signal and idler photons are detected by two NbN superconducting nanowire single photon detectors (SNSPDs)^19^,^20^ after quantum state analyzers for energy-time entanglement or polarization entanglement.

The two SNSPDs are operated at 2.2 K in a Gifford-Mcmahon cryocooler. Since the detection efficiencies of the SNSPDs depend on the polarization of the injected photons, two fiber polarization controllers (FPCs) are used to maximize the detection efficiencies before the two SNSPDs. The detection efficiencies, timing jitters and dark count rates of the two SNSPDs are about 30%, 60 ps and 100 Hz. The detection events of the SNSPDs are recorded by a time correlated single photon counting module (TCSPC, PicoQuant, PicoHarp 300) with a bin width of 4 ps and time resolution of 12 ps rms. The small timing jitters of SNSPDs and high resolution of the TCSPC module ensure that a narrow time domain filter can be utilized in the coincidence measurement to suppress the impact of noise photons [see Methods for more details].

The experiment of Franson-type interference is realized to characterize the energy-time entanglement property, in which two unbalanced Mach-Zehnder interferometers (UMZIs) are used as the quantum state analyzer as shown in Fig. 2(b). The UMZIs are commercial DPSK demodulators (Kylia mint-1x2-L-2.5 GHz) with arm length differences of Δ*t_UMZI_* = 400 ps in time domain. The relative phase differences between the two arms of the UMZIs can be tuned by the voltages applied on the DPSK modulators, which are denoted by *α* and *β*, respectively. According to the FWHM of filters for signal and idler photons, the coherence time of the single photon wave packet of generated photons is about *τ*_1_ ~ 8 ps. On the other hand, the coherence time of the two-photon wave packets generated by the vector SFWM processes is determined by the coherence times of the two pump lights. Since the linewidth of one pump light is far larger than that of the other, the coherence time of the two-photon wave packets can be estimated by the coherence time of the pump light with wider linewidth, which is about *τ*_2_ ~ 0.2 μs. Hence, a Franson-type interference with high visibility can be expected under the condition of 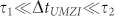.

**Figure 3:**
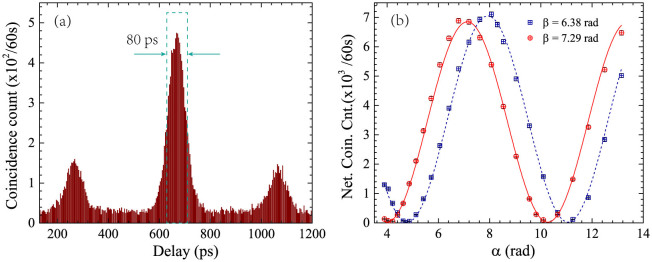
Franson-type interference induced by the energy-time entanglement. (a) A typical result of the coincidence in the experiment of Franson-type interference. The square with dashed line indicates the time domain filter used to extract the coincidence counts. (b) The interference fringes of the net coincidence counts in the experiment of Franson-type interference. The visibilities are 99 ± 1% (*β* = 6.38 rad), 99 ± 1% (*β* = 7.29 rad), calculated by curve fitting.

The experiment results for the energy-time entanglement are shown in Fig. 3. Figure 3(a) is a typical result recorded by the TCSPC in the coincidence measurement, under the single side count rates of 3.77 × 10^5^ Hz and 3.38 × 10^5^ Hz for signal and idler photons, respectively (the insertion losses of the UMZIs for the signal and idler photons are 3.89 dB and 4.16 dB, respectively). There are three coincidence peaks due to the arm length differences of the two UMZIs at signal and idler sides. The central coincidence peak is contributed by two cases, corresponding to that both photons pass through the long arms of the UMZIs and that both photons pass through the short arms of the UMZIs, respectively. The two cases are indistinguishable and their states are coherently superposed, showing the property of energy-time entanglement^16^. As a result, the central coincidence peaks show an interference fringe under varying relative phase differences of the UMZIs. The measurement results of the interference fringes are shown in Fig. 3(b). The raw coincidence counts are calculated by the sum of 20 time bins, equivalent to a time domain filter of 80 ps, which is the FWHM of the coincidence peak. The counting time for each measurement is 1 minute. The accidental coincidence counts are calculated by the sum of the counts in 20 time bins outside the coincidence peaks. The net coincidence counts are obtained by subtracting the accidental coincidence counts from the raw coincidence counts. Figure 3(b) shows the interference fringes of the net coincidence counts, in which the coincidence counts are measured under different *α* when *β* is fixed at 6.38 rad or 7.29 rad, respectively. The interference visibilities are calculated by curve fitting. The interference visibilities for net coincidence counts are 99 ± 1% for both *β* = 6.38 rad and *β* = 7.29 rad. For the raw coincidence counts, the interference visibilities are 84 ± 1% for both *β* = 6.38 rad and *β* = 7.29 rad. During the measurement, the single side count rates are almost unchanged under different relative phase differences of the two UMZIs, showing that the fringes are induced by the energy-time entanglement rather than the interference of single photons. Either the measured visibilities for the net coincidence counts or that for the raw coincidence counts are higher than 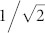, which is the benchmark for the violation of the Bell inequality^21^, indicating the energy-time entanglement generation. Comparing with previous works about the energy-time entanglement^13^,^21^, the raw visibilities are limited mainly due to the noise photons generated by the spontaneous Raman scattering in optical fibers. The ways to improve the raw visibilities are discussed in the section of Discussion.

**Figure 4:**
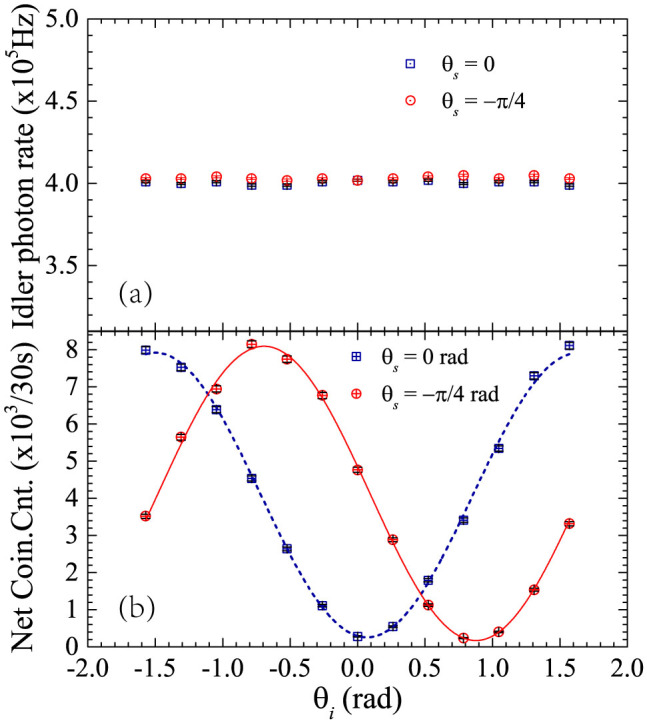
Two-photon interference under two non-orthogonal polarization bases induced by polarization entanglement. (a) The single side count rates of signal photons under *θ_s_* = 0 or *θ_s_* = −*π*/4 rad. (b) The interference fringes of the net coincidence counts. The interference visibilities are 94 ± 1% (*θ_s_* = 0) and 96 ± 1% (*θ_s_* = −*π*/4 rad), calculated by curve fitting.

The entanglement property on the DOF of polarization is explored by the two-photon interference under two non-orthogonal polarization bases, using two polarization analyzers as the quantum state analyzer, which is shown in Fig. 2(c). Each polarization analyzer includes a fiber polarization controller (FPC) and a rotatable polarizer (P). The angles of the polarizers for signal and idler photons are denoted by *θ_s_* and *θ_i_*, respectively. Firstly, *θ_s_* is set to 0 and *θ_i_* is set to *π*/2 rad, then the fiber polarization controllers are adjusted to maximize the coincidence counts. By this process, the detecting directions of the two polarization analyzers are collimated. Figure 4(a) is the single side count rates of signal photons when *θ_i_* is varied and *θ_s_* is set to 0 or −*π*/4 rad. It is shown that the single side count rates are almost independent on the angle of polarizer, indicating that photons at single sides are not polarized in some specific direction. Figure 4(b) is the results of net coincidence counts obtained under a time domain filter of 80 ps. The counting time for each measurement is 30 seconds. It can be seen that the two-photon interference can be observed under two non-orthogonal polarization bases, with fringe visibilities of 94 ± 1% under *θ_s_* = 0 and 96 ± 1% under *θ_s_* = −*π*/4 rad. For the raw coincidence counts, the visibilities are 88 ± 1% under *θ_s_* = 0 and 89 ± 1% under *θ_s_* = −*π*/4 rad, respectively. Both the measured visibilities for the net coincidence counts and that for the raw coincidence counts are higher than 71%^22^, indicating the polarization entanglement of the generated two-photon state.

### Hyper-entanglement in energy-time and discrete-frequency

**Figure 5:**
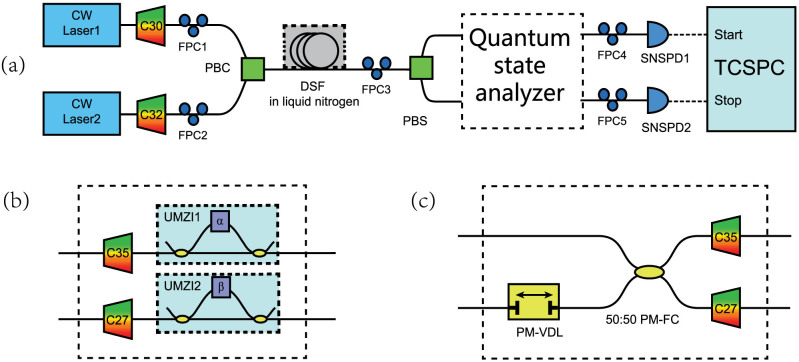
(a) The experiment setup for hyper-entanglement in energy-time and frequency. The two photons are routed to two paths according to their polarization states. (b) The quantum state analyzer for the energy-time entanglement. It is composed of two UMZIs after the optical filters for signal and idler photons; (c) The quantum state analyzer for the spatial quantum beating induced by frequency entanglement. A polarization-maintaining variable delay line (PM-VDL) is used at one arm to adjust the arrival time difference of the two photons at the 50:50 polarization-maintaining fiber coupler (50:50 PM-FC). Two optical filters at signal and idler frequencies are set at the two output ports of the fiber coupler, respectively.

The experiment setup for the demonstration of hyper-entanglement in energy-time and frequency is shown in Fig. 5(a). Comparing with Fig. 2(a), the two setups are similar, however, the optical filter module for separating signal and idler photons in Fig. 2(a) is replaced by a polarization beam splitter (PBS) in Fig. 5(a), which 8separates the generated photons by polarization. The quantum state analyzers for energy-time entanglement and frequency entanglement are shown in Fig. 5(b) and (c), respectively.

**Figure 6:**
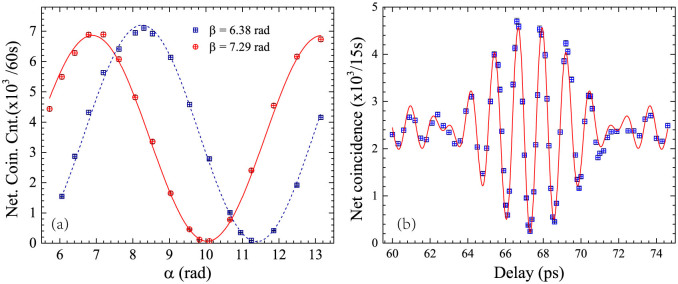
Quantum interference induced by hyper-entanglement in energy-time and discrete-frequency. (a) The interference fringes of the net coincidence counts in the experiment of Franson-type interference. The interference visibilities are 98 ± 1% (*β* = 6.38 rad) and 99 ± 1% (*β* = 7.29 rad), respectively. (b) The spatial quantum beating fringe of the net coincidence counts induced by the frequency entanglement. The visibility of the spatial quantum beating fringe is 91 ± 3%.

The energy-time entanglement is also demonstrated by the experiment of Franson-type nonlocal interference. The quantum state analyzer for this experiment is similar with the one shown in Fig. 2(b), except that two optical filters at signal and idler frequencies are inserted before the UMZIs to suppress the noise photons. The experimental methods and conditions are also similar with the ones in previous experiment and the results are shown in Fig. 6(a). It can be seen that the interference visibilities for the net coincidence counts are 98 ± 1% under *β* = 6.38 rad and 99 ± 1% under *β* = 7.29 rad, respectively. For the raw coincidence counts, the interference visibilities are 91 ± 1% for both *β* = 6.38 rad, and *β* = 7.29 rad. Either the measured visibilities for the net coincidence counts or that for the raw coincidence counts are higher than 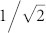, indicating the energy-time entanglement generation.

To demonstrate the discrete-frequency entanglement property, an experiment of spatial quantum beating is realized utilizing the quantum state analyzer shown in Fig. 5(c). The two photons entangled in frequency are injected to a 50:50 polarization-maintaining fiber coupler (50:50 PM-FC). A polarization-maintaining variable delay line (PM-VDL) is inserted in one input port of the fiber coupler to adjust the arrival time difference between the two photons. Two optical filters at signal and idler frequencies are set at the two output ports of the 50:50 PM-FC, respectively.

**Figure 7:**
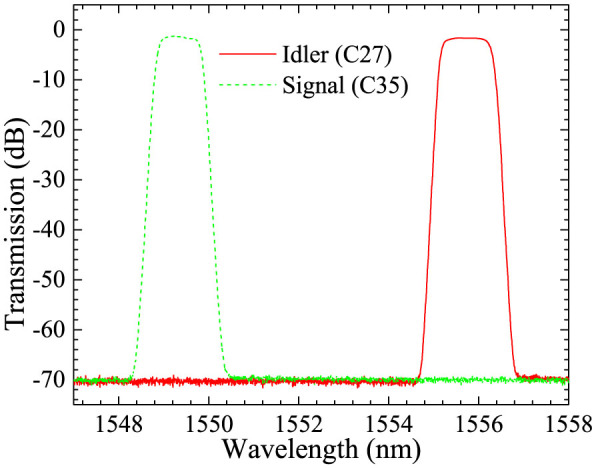
The transmission spectra of optical filters for the signal and idler photons. The measured sideband isolations are about −70 dB, which are limited by the dynamical range of the swept test system (Santec, STS510).

The experiment results of the spatial quantum beating are shown in Fig. 6(b). The squares are measured net coincidence counts under different delay times of the VDL. The solid line is the fitting curve according to^17^
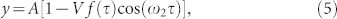
where *τ* is the delay times between signal and idler photons at the 50:50 PM-FC, *V* is the visibility of the spatial quantum beating fringe. *ω*_2_ is the frequency difference between the signal and idler photons. *f*(*τ*) is a normalized function determined by the transmission spectra of the optical filters for the signal and idler photons. In the experiment, the optical filters have flat top and steep falling edges (See Fig. 7 in Method). Hence, their transmission spectra can be approximated by a rectangular function with a bandwidth of *ω*_1_, while, *f*(*τ*) can be approximated by a sinc function, *f*(*τ*) = sin(*ω*_1_*τ*)/*ω*_1_*τ*. Figure 6(b) shows that the fitting curve agrees well with the experiment results of spatial quantum beating. The fitted results are *ω*_1_ = 2*π* × 112 GHz and *ω*_2_ = 2*π* × 788 GHz. Both of them are close to the values calculated by the transmission spectra of the optical filters for the signal and idler photons, which are *ω*_1,0_ = 2*π* × 118 GHz and *ω*_2,0_ = *ω_s_*_0_ − *ω_i_*_0_ = 2*π* × 800 GHz, respectively. The differences between the fitting results and the calculated values may be due to that the two pump frequencies (*ω_p_*_1_ and *ω_p_*_2_) and central frequencies of the optical filters for signal and idler photons (*ω_s_*_0_ and *ω_i_*_0_) have a small mismatch. The interference visibility is *V* = 91 ± 3% for net coincidence counts. For the raw coincidence counts, the visibility is 85 ± 3%. According to Ref. 23, the nonzero visibility of spatial quantum beating in our experiment indicates the discrete-frequency entanglement property in the generated photon pairs. Comparing with previous works of frequency entanglement^24^, the net visibility of spatial quantum beating in this paper is mainly limited by the polarization misalignment at the PBS or 50:50 PM-FC.

## Discussion

In the experiments shown in this paper, although the interference visibilities for the raw coincidence are all higher than the criterion for entanglement, they are limited by noise photons mainly generated by the spontaneous Raman scattering. In our experiments, two methods have been applied to suppress the impacts of the spontaneous Raman scattering. On one hand, the optical fiber is cooled to 77 K by liquid nitrogen bath to reduce the Raman noise photon generation rates^18^. On the other hand, narrow time domain filters are used to reduce the contribution of the noise photons to the coincidence counts [see Methods for more details]. However, since the raw fringe visibilities indicate the performances of the proposed hyper-entanglement generation scheme in quantum information applications, higher raw fringe visibilities are always desired. A direct way to increase the raw fringe visibility is cooling the fiber to lower temperature. Previous works of fiber based quantum light sources have shown that Raman noise photon generation rates can be highly reduced by cooling the fiber to 4 K through liquid helium bath^25^. Recently, photon pair generations in on-chip integrated waveguides, such as silicon waveguides^26^,^27^ or chalcogenide waveguides^28^, have attracted much attention. Quantum light sources based on these waveguides have the potential for room temperature operation thanks to their properties of low Raman noises. Hence, the scheme proposed in this paper provides a way to realize on-chip integrated hyper-entanglement sources with high performance, if proper waveguide designs are made to support the two vector SFWM processes in these waveguides.

It is worth noting that when the two pump lights propagate through the optical fiber, scalar SFWM processes by single pump light would also be stimulated. Since the frequency relations among the pump lights and generated signal and idler photons in the scalar processes are different from those of vector SFWM processes, the scalar processes have no contribution on the hyper-entanglement generation if proper optical filters are used to select signal and idler photons. Hence, in this scheme the scalar SFWM processes only introduce noise photons at both sides without correlation, like the noise photons generated by the spontaneous Raman scattering. Our previous work^13^ about energy-time entanglement generation in optical fibers has shown that the photon generation rate by the scalar SFWM processes under CW pumping is far smaller than the noise photon generation rate by the spontaneous Raman scattering. Furthermore, the impact of noise photons can be effectively suppressed by the technique of time domain filters [see Methods for details]. Hence, the scalar SFWM processes in the fiber have little impact in this scheme.

As a summary, we proposed and demonstrate a generation scheme for telecom band hyper-entanglement based on vector SFWM processes, which are stimulated by two orthogonal polarized CW pump lights with different frequencies. Two kinds of hyper-entangled two-photon states are generated. One is hyper-entangled in DOFs of energy-time and polarization. Its property of energy-time entanglement is demonstrated by the Franson-type interference with fringe visibilities (for the net coincidence counts) of 99 ± 1% for both *β* = 6.38 rad and *β* = 7.29 rad. Its property of polarization entanglement is demonstrated by the two-photon interference under two non-orthogonal polarization bases, with fringe visibilities (for net coincidence counts) of 94 ± 1% under *θ_s_* = 0 and 96 ± 1% under *θ_s_* = −*π*/4 rad, respectively. The other is hyper-entangled in DOFs of energy-time and discrete-frequency. The property of energy-time entanglement is also demonstrated by the Franson-type interference, with fringe visibilities (for net coincidence counts) of 98 ± 1% under *β* = 6.38 rad and 99 ± 1% under *β* = 7.29 rad, respectively. Its property of frequency entanglement is demonstrated by the experiment of spatial quantum beating, with a fringe visibility (for net coincidence counts) of 91 ± 3%. This scheme provides a simple way to realize telecom band hyper-entanglement, which has potentials for long distance distribution over optical fiber, preferred in large geographic-scale applications of quantum communication and quantum information.

## Methods

### Optical filtering for signal photons, idler photons and pump lights

In the experiments, the optical filters for signal photons, idler photons and pump lights are realized by cascading 4 DWDMs, respectively, which are commercial devices for optical communication. The insertion losses of these filters are all lower than 2 dB. Their isolations at adjacent channels are larger than 120 dB, estimated by the parameters of each DWDM device and the fact that commercial DWDM devices have very high return loss. For non-adjacent channels, their isolations are even much higher. The transmission spectra of the filters for signal and idler photons are shown in Fig. 7, measured by a swept test system (Santec, STS510).

### Noise suppression by the narrow time domain filter

In the experiment, under the CW pumping configuration, the generation rate of noise photons in optical fibers, including those by the spontaneous Raman scattering and those by the scalar SFWM processes, is much higher than that of entangled photon pairs by vector SFWM processes. A narrow time domain filter is applied in the experiment to suppress the impact of noise photons. To measure the coincidence counts and accidental coincidence counts, the detection events of the two SNSPDs are delivered to the trigger port and signal port of the TCSPC, respectively. The event sent to the trigger port starts the timer in the TCSPC. The event sent to the signal port stops the timer in the TCSPC, forming a start-stop event. In the measurement, a histogram is carried out by the statistics on the spans of recorded start-stop events. If the start-stop event is generated by an entangled photon pair, its span should be a fixed value, which is determined by the travel time difference of the two photons to the two SNSPDs. On the other hand, the spans of the start-stop events due to noise photons are random in time. Hence, the contribution of entangled photon pairs can be discriminated in the histogram and selected out by a time domain filter. It can be expected that the narrower the time domain filter, the better the noise suppression, however, the peak for the coincidence counts would be extended by the timing jitters of single photon detectors and the TCSPC module, which provides a limit to the filter width. Comparing with the single photon detectors based on APDs (Avalanche photodetectors), SNSPDs have far lower timing jitters. In the experiment, the timing jitters of the two SNSPDs are 60 ps, respectively, and the timing jitters of the TCSPC is 12 ps. Such low timing jitters of SNSPDs and the TCSPC support a time domain filter width as narrow as 80 ps, leading to a high suppression of the impact of noise photons on coincidence count measurement.

## Author Contributions

S.D. designed and performed experiments, analyzed data and wrote the manuscript. L.J.Y., J.J.W. and W.J.Z. performed experiments. W.Z. designed experiments, analyzed data, wrote the manuscript and supervised the project. L.X.Y. and Y.D.H. supervised the project and edited the manuscript.
